# The Rbf1, Hfl1 and Dbp4 of *Candida albicans* regulate common as well as transcription factor-specific mitochondrial and other cell activities

**DOI:** 10.1186/1471-2164-15-56

**Published:** 2014-01-22

**Authors:** Kasra Khamooshi, Patricia Sikorski, Nuo Sun, Richard Calderone, Dongmei Li

**Affiliations:** 1 Department of Microbiology & Immunology, Georgetown University Medical Center, Washington DC 20057, USA

**Keywords:** Transcription factor, Non-glucose carbon metabolism, Mitochondria, Lipid oxidation, Metabolic regulation, *Candida albicans*

## Abstract

**Background:**

Our interest in *Candida albicans* mitochondria began with the identification of *GOA1*. We demonstrated its role in cell energy production, cross-talk among mitochondria and peroxisomes, non-glucose energy metabolism, maintenance of stationary phase growth, and prevention of premature apoptosis. Its absence results in avirulence. However, what regulated transcription of *GOA1* was unknown.

**Results:**

To identify transcriptional regulators (TRs) of *GOA1*, we screened a *C. albicans* TF knockout library (TRKO) and identified Rbf1p, Hfl1p, and Dpb4p as positive TRs of *GOA1*. The phenotypes of each mutant (reduced respiration, inability to grow on glycerol, reduced ETC CI and CIV activities) are reasonable evidence for their required roles especially in mitochondrial functions. While the integration of mitochondria with cell metabolic activities is presumed to occur, there is minimal information on this subject at the genome level. Therefore, microarray analysis was used to provide this information for each TR mutant. Transcriptional profiles of Rbf1p and Hfl1p are more similar than that of Dpn4p. Our data demonstrate common and also gene-specific regulatory functions for each TR. We establish their roles in carbon metabolism, stress adaptation, cell wall synthesis, transporter efflux, peroxisomal metabolism, phospholipid synthesis, rRNA processing, and nuclear/mtDNA replication.

**Conclusions:**

The TRs regulate a number of common genes but each also regulates specific gene transcription. These data for the first time create a genome roadmap that can be used to integrate mitochondria with other cell processes. Of interest, the TRs are fungal-specific, warranting consideration as antifungal drug targets.

## Background

Fungal invasive infections of humans are now referred to as “hidden killers” [1]. More than 90% of these infections are caused by species of *Candida*, *Cryptococcus*, *Aspergillus*, and *Pneumocystis*[1]. Blood-borne, nosocomial candidiasis is ranked 4^th^ in frequency in the USA with a crude and attributable mortality of 49 and 27% (USA), similar to other developed countries [1,2]. The incidence of candidiasis has increased sharply over the past few decades primarily due to cancer chemotherapy, organ/bone marrow transplantation, surgical intervention, and the AIDS pandemic [3,4]. Treatment of these infections costs ~ $2.0-2.6 billion per year [5-8]. Global cryptococcal meningitis (usually caused by *C. neoformans*) in HIV/AIDS patients and others with immunosuppression therapies is estimated at 1 million cases per year; 620,000 deaths alone are in Sub-Saharan Africa [1]. *Cryptococcus gattii* is an emerging pathogen of apparently healthy people, signifying its potential as an even more dangerous invasive fungus. Death from the top 10 invasive fungi (1-1.5 million) is equal to or more than that of tuberculosis (1.4 million) or malaria (971,000) [1]. The enormity of fungal infections is magnified by the non-invasive (superficial) infections such as nail and scalp infections (1.9 billion), vaginal infections of women during child-bearing years (frequency of 50-75%), and oral and esophageal candidiasis in HIV/AIDS patients (12 million). In part, the rising costs are associated with inappropriate therapy, defined as delayed intervention, inadequate dosage, or administration of an antifungal to which an isolate was considered drug resistant [6].

*C. albicans* remains as the most common cause of candidiasis among all *Candidia* species. Virulence of this organism is commonly attributed to factors that initiate colonization of host cells (the ALS gene family and others), cause invasion (secreted lipases and proteases), regulate morphogenesis (the yeast hyphal transition), and biofilm formation [1]. *In vivo* virulence of these factors has been established in animal models fulfilling the paradigm of “Molecular Koch’s postulates”. Aside from the construction of single mutants to verify a role in pathogenesis, another useful approach to understanding virulence is to characterize global gene differences between a pathogen (*C. albicans*) and a non-pathogen (*Saccharomyces cerevisiae*, model yeast) or between two pathogens, one with a much lower incidence of causing candidiasis (*C. dublinensis*) [9]. Both types of data suggest interpretations of the gene repertoire needed by a pathogen. One of the major differences between *C. albicans* and model yeast is a rewiring of transcriptional regulation [10]. For *C. albicans*, enzymes of alternative carbon metabolism (non-glucose substrates) are stabilized even in the presence of glucose, compared to model yeast of which these same enzymes are regulated by glucose-repressible events [11]. Speculation is that *C. albicans* maintains a backup source for energy and carbon conservation to respire when confronted with low levels of host glucose. Model yeast when grown aerobically uses glucose via glycolysis and is referred to as Crabtree-positive. Oppositely, *C. albicans* respires oxidatively in the presence of glucose and is Crabtree-negative [12]. These observations are not surprising, given the differences in their environmental niches. In the case of *C. albicans*, low blood levels of glucose cause the utilization of alternative carbon sources as mentioned above and described in other labs [13-16]. Some peroxisomal activities in *C. albicans* are critical to the pathogenesis of candidiasis, since these organelles house alternative carbon metabolic pathways (such as the glyoxylate cycle) that are critical to survival of the organisms in macrophages [15].

Our interest in mitochondria of *C. albicans* began with the identification of *GOA1*[16]. Functional annotation was developed based upon phenotypic assays of a *goa1*Δ null mutant. Goa1p translocates to mitochondria during stress and in the presence of non-glucose substrates such as glycerol. The protein regulates complex I (CI) of the electron transport chain and also interacts with peroxisomes, organelles that house alternative carbon metabolic pathways. The loss of *GOA1* causes a major reduction in mitochondrial membrane potential and a concomitant reduction in the formation of ATP. We have shown that a dysfunctional CI causes an increase in reactive oxidant species (ROS), triggering apoptosis and an associated shortened chronological aging *in vitro*[16-18]. We demonstrated that cross-talking between mitochondria and peroxisomes in the presence of either glucose or non-glucose substrates requires Goa1p [17,19]. Importantly, there are several defects in the mutant in regard to virulence and host cell interactions [20]. Compared to parental and gene-reconstituted strains, *goa1*Δ is avirulent in a murine model of blood-borne candidiasis, killed readily by human neutrophils *ex vivo*, and hypersusceptible to triazoles. Microarray analyses of *goa1*Δ indicate a major down regulation of genes associated with peroxisomal functions and carbon metabolism [17,19]. But, what regulates *GOA1*? Herein, we report on the identification of three transcription regulator (TR) Rbf1p, Hfl1p, and Dpb4p, each of which positively regulates *GOA1*. Importantly, we assign functions to each of these transcription factors in the regulation of cellular processes. Two of these TRs have not been functionally annotated, and the third (Rbf1p) is a repressor of filamentation [21,22]. Using microarray analysis, we show that there is both TR-specific gene regulation as well as regulation of a set of common genes.

## Results

### Identification of transcription regulators of *GOA1*

**Figure 1:**
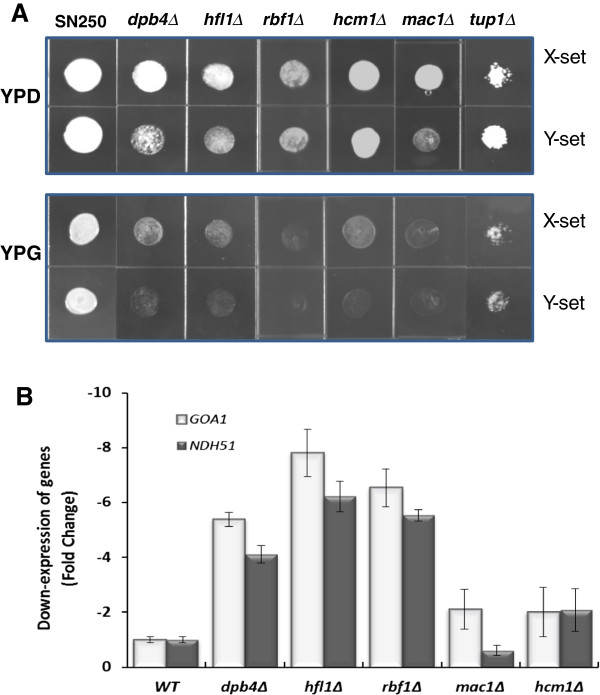
**Glycerol utilization and transcriptional regulation of *GOA1 and NDH51 *by the TRKOs. A**. Drop plate assays of 6 TRKO mutants in YP-dextrose (YPD) or YP-glycerol (YPG). Each mutant was spot inoculated with 3 μl of stock cells and grown at 30°C for 24 h. X- and Y-sets refer to two independently constructed mutants lacking each gene. The reduction or lack of growth on YPG indicates a mitochondrial respiratory dysfunction. **B**. Each of the mutants shown in (A) was measured for transcription of *GOA1* by Real-time PCR. Data are indicated as a down regulation of either *GOA1* or *NDH51*. The latter encodes a Complex I subunit of the mitochondrial electron transport chain. Dpb4p, Hfl1p and Rbf1p are positive regulators of both *GOA1* and *NDH51*.

To initially identify transcription factors that regulate *GOA1*, we screened a transcription regulator knockout (TRKO) library of *C. albicans*[21]. The library was maintained in 96-well microtiter plates at -80°C. The initial screen of 163 TR mutants was done in 36-well plates containing YP-dextrose (YPD) or YP-glycerol (YPG), since glycerol is only a substrate for mitochondrial oxidation. Cultures of each mutant were grown overnight and inoculated in each medium. We identified 6 mutants that were either unable to grow or grew poorly only on YP-glycerol (YPG) (Figure 1A and Table 1). Two independently generated mutants of each gene were used in assays for growth on both YPD and YPG (Figure 1A). Each independent gene mutant is identified as X and Y.

**Table 1:** TRKOs those are unable to grow on YP-Glycerol and their mutant phenotypes

**ORF**	**Gene**	**Description**	**Mutant phenotype**
orf19.4853	*HCM1*	Has a forkhead domain; involved in hyphal morphogenesis; similar to the *S.cerevisiae* ortholog that regulates transcription during S-phase of the mitotic cell cycle	Vegetative growth decreased in YPG
orf19.7068	*MAC1*	Regulon of *CaCTR*1, a high affinity copper transporter gene; role in ion homeostasis	Vegetative growth decreased in YPG
orf19.2088	*DPB4*	Putative DNA polymerase epsilon subunit D; Ortholog of *S.cerevisiae* involved in DNA-dependent DNA replication	Viable, slow-growing and displays abnormal invasive growth
orf19.3063	*HFL1*	HAP5-like; phosphorylated protein	Vegetative growth decreased in YPG
orf19.5558	*RBF1*	Glutamine-rich activation domain; binds RPG-box DNA sequences; antigenic during human oral infection	Vegetative growth decreased in YPG
orf19.6109	*TUP1*	Co-repressor; represses filamentous growth; regulates switching	Abnormal colony appearance on YPD and YPG

The mutants that did not grow on YPG were assayed for transcription of *GOA1* using real-time PCR (Figure 1B). Of the six mutants, we observed significant down regulation of *GOA1* in mutants lacking *HFL1*, *RBF1*, or *DPB4*. Transcription levels for both *GOA1* and *NDH51* (Ndh51p is a complex I subunit protein) were decreased 4-6 fold in *RBF1*, *HFL1,* and *DPB4* TRKO strains. Orf19.2088 is named *DPB4* which corresponds to the *S. cerevisiae* ortholog. The *MAC1* and *HCM1* mutants had much smaller changes than the *RBF1*, *HFL1,* or *DPB4* TRKO strains *vs.* wild type (WT) cells (Figure 1B). The knockout strain for *TUP1* was excluded from further studies because of its poor viability. *HCM1* and *MAC1* are conserved transcriptional regulators in *C. albicans* and *S. cerevisiae*[21]. Mac1p and Tup1p are thought to regulate copper and iron uptake [23]. Our data indicate that we have identified three TRs that regulate *GOA1* and *NDH51* both of which are required for mitochondrial activity in *C. albicans*. Of importance, their role as regulators of cell metabolism has not been described. The remaining sections reflect our studies of the 3 TR mutants whose transcription of *GOA1* was down regulated.

### Morphology and growth of *hfl1Δ*, *rbf1Δ*, and *dpb4Δ*

**Figure 2:**
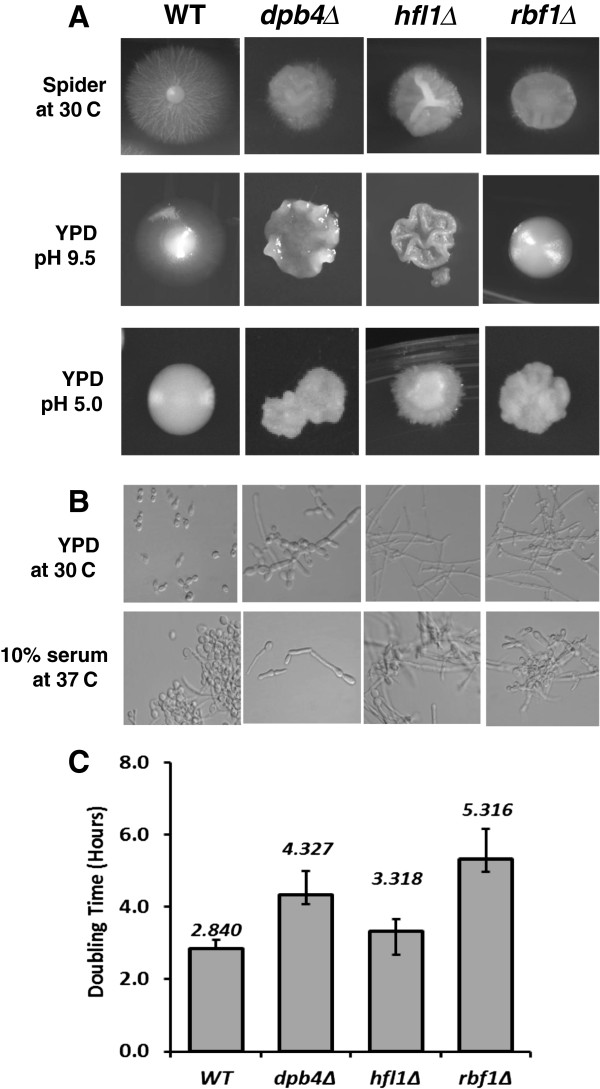
**The yeast to hyphal transition, generation time, and transporter activity are altered in each of the TRKO mutants. A**. Growth on Spider agar, YPD pH 9.5 and YPD pH 5.0. On Spider agar, all mutants had reduced colony growth. On YPD (below), colony diameter was again reduced and each mutant had a wrinkled colony appearance with a lack of filamentation at pH 9.5. **B**. Microscopic growth of each mutant is shown. In YPD (30°C), each of the TRKO mutants was constitutively filamentous compared to the yeast growth of WT cells (SN250) as reported previously [21,22]. In 10% serum, all mutants and WT strains are filamentous.** C**. Doubling times are shown for WT (SN250) as well as each TRKO strain. Strains *dpb4*∆ and *rbf1*∆ were significantly higher than WT cells. Strain *hfl1*∆ was not significantly different than WT cells. Doubling time was determined by cell mass measurements.

A comparison of morphology as well as doubling times was carried out with the *RBF1, HFL1* and *DPB4* TRKO strains (Figure 2,A-C and Table 1). Abnormal colony phenotypes (Figure 2A) and filamentous microscopic growth (Figure 2B) were observed in most growth conditions compared to the parental strain. Each of the three TRKO strains exhibited smaller colonies and a near absence of filamentous rings at the edges of colonies compared to WT colonies on Spider medium (Figure 2A). The surface of colonies from mutant strains was extensively wrinkled by day 7 on both Spider and YPD (pH 9.5) agar media. Invasive growth on YPD-2% glucose, pH 9.5, also was diminished in the three TRKO strains. All mutants were constitutively filamentous in YPD at 30°C. However, *dpb4*∆ was less filamentous compared to the other two mutants (50% of cells), and those were mostly pseudohyphae. *Rbf1*Δ and *hfl1*Δ were similar to WT cells in 10% serum at 37°C, as reported previously for *rbf1*Δ [21]. The growth phenotypes of each mutant are summarized in Table 1 and also described at the *Candida* CGD database (http://www.candidagenome.org). Doubling times varied according to the specific mutant from 3.32 to 5.32 hr compared to WT cells (2.84 hours) (Figure 2C).

### Antifungal susceptibilities of the TR mutants

Since we showed previously that deletions of *GOA1* and *NDH51* resulted in hypersensitivity to triazole antifungals [19], we compared the susceptibilities of each TR mutant to the antifungals fluconazole, amphotericin B (AmB), and caspofungin (Table 2). Since trailing has been reported, we measured both MIC_50_ and MIC_100_ for fluconazole. Similar to our published data on *goa1*Δ and *ndh51*Δ, *rbf1Δ* and *hfl1Δ* are hypersusceptible to 1-2.0 μg/ml fluconazole (MIC_100_), while *dpb4*Δ was similarly susceptible as parental cells. The susceptibilities of all mutants to AmpB and caspofungin were not statistically different compared to parental cells.

**Table 2:** Antifungal drug susceptibilities to cell membrane and cell wall inhibitors*

**Gene KO**	**Fluconazole(μg/ml)**		**AmB (μg/ml)**	**Caspofungin (μg/ml)**
	**MIC**_**100**_	**MIC**_**50**_	**MIC**_**100**_	**MIC**_**100**_
**WT**	256	0.5	0.25	0.25
***GOA1***	1.0	1.0	1.0	0.0625
***DPB4***	256	1.0	1.0	0.5
***HFL1***	2.0	0.25	0.5	0.125
***RBF1***	2.0	0.25	0.25	0.0625

*Data reflect averages of 3 experiments.

### The TF mutants are hypersusceptible to inhibitors of cell wall formation

**Figure 3:**
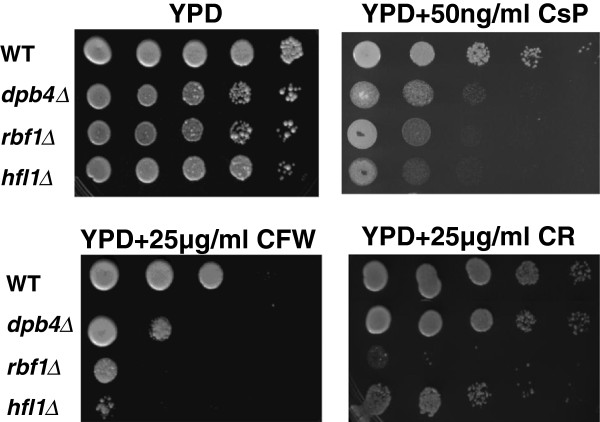
**The TRKO mutants (indicated on the left) are susceptible to the cell wall inhibitors calcofluor white, Congo red and caspofungin.** Cell wall inhibitors are YPD only (control), YPD + caspofungin (CsP) first row; YPD + calcofluor white (CFW) or + Congo Red (CR) in the second row. Compared to growth on YPD only, each mutant was hypersensitive to CFW and caspofungin, while only *rbf1*∆ was hypersensitive to CR. The *hfl1*∆ displayed a slight sensitivity to CR.

Surprisingly, all mutants were hypersusceptible to calcofluor white (CFW) and caspofungin on drop plate assays, but *rbf1Δ*and *hfl1Δ* were more so than *dpb4*Δ (Figure 3). As for susceptibility to Congo red (a β-1,3-glucan inhibitor), only the *rbf1Δ* and to a lesser extent *hfl1Δ* were more susceptible than WT cells*.* Thus, *rbf1*Δ was affected most by cell wall formation inhibitors. The differences between the caspofungin MICs (described above) and cell wall inhibitor drop plate assays suggest that the regulation of cell wall integrity among the three TRs is different. However, the disparities of both assays could also be an explanation as MIC determinations were done using RPMI medium at 37°C while drop plate assays were done in YPD agar at 30°C.

### Mitochondrial defects in *rbf1Δ, hfl1Δ and dpb4Δ*

**Figure 4:**
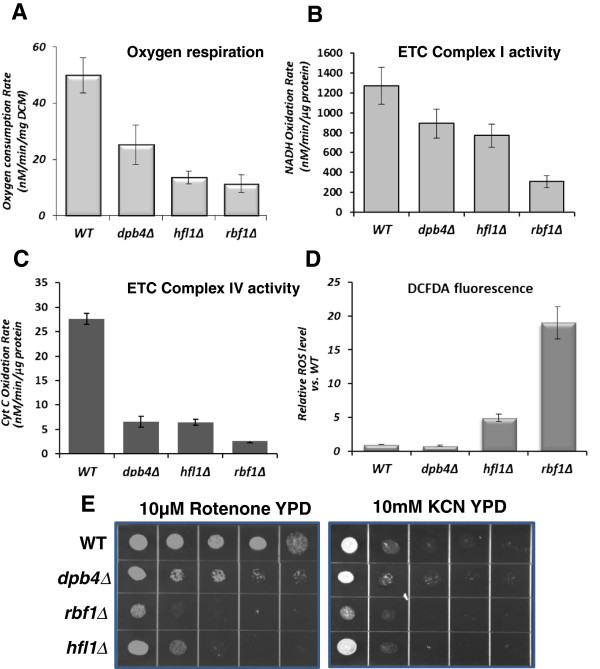
**The TRKO mutants are deficient in respiratory functions. A**. respiration; **B**. ETC CI activity; **C**. ETC CIV activity, and **D**. ROS production.*dpb*4∆, *hfl1*∆, and *rbf1*∆ each respired less (2.2-5-fold reduction) than WT cells. CI activity decreased in each mutant proportionally to their decrease in respiration. CIV activity was 5.5 fold lower in the TRKO mutants. In **D**, ROS production compared to WT cells was highest in the *rbf1*∆, although also significantly increased in *hfl1*∆. The *dpb4*∆ produced ROS equal to WT cells. **E**. ETC CI and CIV inhibitors**:** growth of mutants and WT cells on YPD containing either rotenone (C1 inhibitor) or KCN (CIV inhibitor) is shown. *rbf1*∆ and *hfl1*∆ are hypersensitive to both inhibitors while *dpb4*∆ was less so.

The inability of each mutant to assimilate a non-fermentable carbon source (glycerol) indicates defects in mitochondrial respiration. Further, we were interested in comparing the functions of each of the TRKO strains to *GOA1* in energy production and carbon metabolism. To determine the mitochondrial status of the TRKOs, we first measured oxygen consumption among mutants and parental cells. The oxygen consumption rates were decreased by 2.2-fold for *dpb4Δ,* and about 5-fold in *hfl1Δ* and *rbf1Δ* compared to WT cells (Figure 4A). For these experiments, total oxygen consumption was determined from equal masses of cells (per mg dry cell mass, DCM).

The ETC CI and CIV activities (Figure 4B, C), reactive oxidant levels (ROS) (Figure 4D, and susceptibilities to ETC CI and CIV inhibitors (Figure 4E) were also evaluated in *rbf1Δ, hfl1Δ and dpb4Δ* compared to WT cells. ETC CI and CIV enzyme activities for the *rbf1Δ* mutant were significantly reduced by 4-fold and 14-fold, respectively. Corresponding to the decrease in CI enzyme activity was an increase in sensitivity to rotenone, a CI inhibitor and KCN (CIV inhibitor) in *rbf1Δ*. For *hfl1Δ,* CI activity was less affected than *rbf1Δ,* but CIV activity was reduced similarly to *rbf1Δ.* CI enzyme activity in *dpb4*Δ was similar to that of *hfl1Δ.* Sensitivity of the *dpb4*∆ to rotenone was less than that of the other 2 mutants but the same as *hfl1*∆ in regard to KCN sensitivity. These data indicate that each of the TR mutants have altered CI and especially CIV enzyme activity although correlates with complex inhibitors are not absolute.

One of the striking features of mitochondria with dysfunctional CI and CIV activities of the ETC is an increase in mitochondrial ROS [17,18]. In this regard, ROS levels were nearly 20-fold higher in *rbf1Δ* and *~*5-fold higher in *hfl1Δ*; however, ROS production in *dpb4Δ* was similar to that of parental cells (Figure 4D), indicating that the ROS scavenging system was less functional in *hfl1Δ* and *rbf1Δ* but not affected in *dpb4Δ.* Microarray data indicated that genes associated with ROS detoxification such as *SOD3, GPX1, GPX2,* in each mutant were increased slightly, but a down regulation in *SOD6* and *GRX1* occurred in both *hfl1Δ* and *rbf1Δ* (Additional file 1: Table S1, Additional file 2: Table S2 and Additional file 3: Table S3). The decrease in *SOD6* and *GRX1* transcription may partially explain the high ROS levels in *hfl1Δ* and *rbf1Δ*.

### Global transcriptional profiling in *rbf1Δ, hfl1Δ,* and *dpb4*Δ

Based upon our published data on transcriptional profiling of the *goa1*Δ [19] and the functions of the *RBF1*, *HFL1*, and *DPB4* as positive regulators of *GOA1*, we expected common gene pools as well as TR-specific gene changes. To obtain data to support this premise, we compared array data from each TR mutant to *goa1*Δ versus their own parental strains. A 2-fold increase/decrease in transcription was used to determine if significant changes occurred.

### General observations of transcriptional changes for each TR mutant

**Figure 5:**
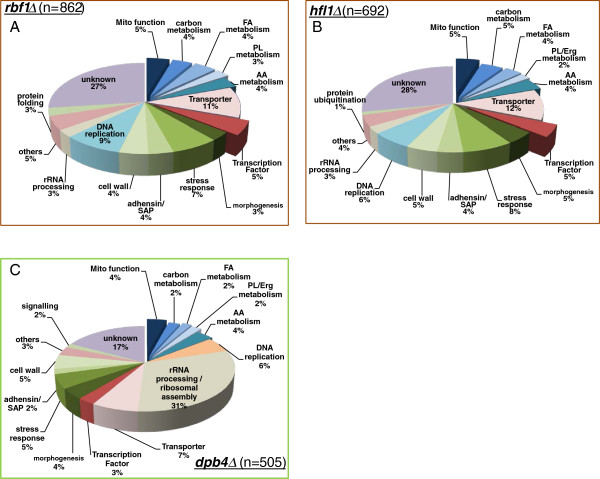
**Total gene changes in the TRKO mutants of *C. albicans rbf1*∆ (A), *hfl1*∆ (B) *dpb4*∆ (C).** Genes with more than a 2-fold or less than 2-fold change in expression levels are included in the diagram. Functional classification of each category is based on the *C. albicans* genome database (http://www.candidagenome.org/) and curated manually with *S. cerevisiae* genome database (http://www.yeastgenome.org/) when needed.

The total number of genes whose transcription changed significantly compared to SN250 was 862 (*rbf1*Δ), 692 (*hfl1*Δ) and 505 (*dpb4*Δ) (Figure 5)*.* The genes with up/down changes in expression *vs.* the parental strain were grouped for each TR mutant based on their functional classification. The assignment of functional categories is based on the information provided by the *C. albicans* CGD and *S. cerevisiae* databases. The% of uncharacterized genes in *hfl1*Δ and *rbf1*Δ was 27-28%, while that of *dpb4*∆ was 17%. We found that 20% of the genes from *rbf1*Δ and *hfl1*Δ were associated with mitochondria, carbon, lipid and amino acid metabolic process. For *dpb4*Δ, the genes of metabolic processes accounted for only 14% (Figure 5).

**Figure 6:**
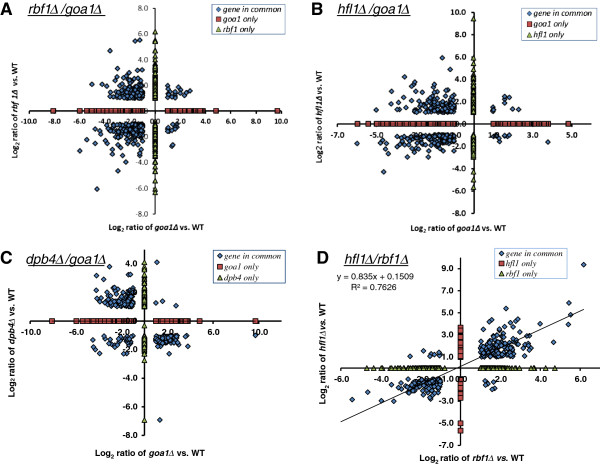
**A comparison of the transcriptomes reveal differences among the 3 TRKO mutants.** Data are derived from *RBF1 ***(A)**, *HFL1 ***(B)**, *DPB4 ***(C)** TRKO strains *vs.* the *GOA1* mutant, and *rbf1 vs. hfl1* mutants **(D)**. Genes common to both the *goa1*∆ and either *rbf1*∆, *hfl1*∆, or *dpb4*∆ are depicted as blue diamonds. The *goa1∆*-specific genes are indicated as red squares, and TRKO-specific genes as green triangles. Upregulated genes for each TR mutant are show above the horizontal axis, down regulated genes below the horizontal axis; while the right side and left side of vertical axis represent upregulated and down regulated genes for *goa1Δ*. Each quadrant reflects a cluster of similar genes between *goa1∆* and TR mutants. **Upper left**: cell wall, stress adaptation, and morphological switching; **lower left**: metabolism; **lower right**: different rewiring responses for common genes, in Figure 6C reflecting DNA replication or maintenance and rRNA processing between *dpb4*∆ and *goa1∆*; **Upper right**: upregulated genes of different functional annotation.

To determine differences among the three TRs in metabolic genes, we compared each TR transcriptome with the *goa1*Δ transcription profile. We found that a *s*ignificant overlap of common genes was observed between *rbf1*Δ and *goa1*Δ (334 in total) and between *hfl1*Δ and *goa1*Δ (302 in total). By contrast, the number of genes common to both *dpb4*Δ and *goa1Δ* was less (247 in total). Gene data are represented in separate sections in Figure 6A-C for each TR mutant in comparison to *goa1Δ* data published previously [19], and Figure 6D compares *rbf1*Δ and *hfl1*Δ. Common down regulated genes encoding putative metabolism functions cluster in the lower left quadrants for the three TR mutants (Figure 6A-C), respectively. Common cell wall, morphology switching, and stress upregulated genes cluster in the upper left quadrants. The group of genes in the lower right quadrant in *dpb4*Δ (Figure 6C) is related to ribosomal /mtRNA processing and DNA/mtDNA replication or maintenance, which is down regulated in *dpb4*Δ. The upper right quadrants for each TR mutant represent a number of transcriptionally altered but non-functionally clustered genes. Green triangles indicate TR-specific genes and red squares indicate *GOA1*-specific genes (Figure 6A-C). The down regulated cluster of genes in *dpb4*∆ only (Y axis of Figure 6C) includes 5 mtDNA genes that encode the ETC CI subunit. The similarity of gene transcriptome changes between *rbf1*Δ and *hfl1*Δ reaches a maximum in the co-linearized rate (R^2^ = 0.76) that is derived from common gene pools in a total of 326 genes (Figure 6D).

There are several points of interest from these data. First, down regulated metabolic genes (left lower quadrants) are much greater in number from *rbf1*Δ (Figure 6A) and *hfl1*Δ (Figure 6B) than in the *dpb*4Δ (Figure 6C). Second, *dpb*4Δ has a much greater number of down regulated genes that are associated with mitochondria DNA and protein synthesis machinery, such as mtDNA encoding genes and mitochondrial rRNA ribosomal assembly (lower right quadrant) compared to the other TRs (Figure 6C, Table 3). As shown in Figure 5, the total% of gene changes in rRNA processing and ribosomal assembly was 31% in *dpb4*∆ compared to 3% in *rbf1*Δ and *hfl1*Δ*.* Third, the cluster size of upregulated genes associated with cell wall integrity, stress responses, morphological switching, and adhesion are reasonably similar for all TRs but opposite with *goa1*Δ (upper left quadrant). These data demonstrate that the number of genes associated with metabolic and other cellular bioprocesses varies among the TR mutants, suggesting differences in their regulatory roles.

**Table 3:** The transcription profiles of mitochondrial functions of TRKOs

**Biological processes**	***rbf1Δ***		***hfl1Δ***		***dpb4Δ***	
	**down**	**up**	**down**	**up**	**down**	***up***
ETC subunits & assembly, alternative oxidase	*orf19.1691*	*NAD5, AOX2*	*NDE1*	*NAD6, MRF1*	*NAD2, NAD3*	*MRF1*
	*ATP18, CYT1*	*orf19.3353*		*COX2*	*NAD4L, NAD4*	
	*QCR8, QCR9*	*orf19.6374*		*orf19.230*	*NAD5, ATP19*	
	*RIP1, orf19.2249*	*MRF1*		*orf19.3353*	*orf19.4273*	
Mitochondrial genome maintenance	*SUV3*	*PRP13,OGG1*	*SUV3,CPA1*		*SUV3, RPP1*	
		*RFC4*	*orf19.3062,RAD32*			
Mitochondrial tRNA processing & ribosome assembly	*orf19.1201*	*MRPL6*	*orf19.1356*		*orf19.2309, orf19.2384*	
	*orf19.1356*					
	*orf19.5161*				*orf19.3303, orf19.4160*	
					*orf19.414, TRM1*	
Mitochondrial carbon metabolism	*GCV2, ALD6*	orf19.850	*UCF1,CTN1*	*orf19.3043*	*IDH2*	
			*orf19.2184, ALD6*	*GDB1,MAL2*		
			*ILV6,DLD1 DLD2,PDK2*			
Mitochondrial transporters	*orf19.4583*	*PAM17, ERV1*	*ATM1,orf19.28*		*RIM2, PET9*	
	*orf19.5129*		*orf19.6532*		*orf19.5628*	
	*orf19.6555*		*orf19.6555 orf19.4966*		*orf19.28*	
			*MDL2,FRP5*			
			*orf19.3455*			
Others	*ISA2*	*orf19.2401*	*orf19.2175*	*PST2*		
	*orf19.2749*	*orf19.2544*	*orf19.6736*			
	*orf19.6550*	*orf19.2825, orf19.4523*	*GTT12*			
	*orf19.5270*	*orf19.4612, orf19.6283*				

### Functional clustering of gene changes in *rbf1Δ, hfl1Δ,* and *dpb4*Δ

#### ***Mitochondrial respiration***

As shown in Figure 4A, oxygen consumption was reduced by 5-fold of WT levels in the *hfl1*Δ and *rbf1*Δ and by 2.2-fold in *dpb4*Δ. This observation is consistent with their increased doubling times *in vitro* in Figure 2C, while also indicating some degree of dysfunctional mitochondria. To better understand the reasons for the reduction in respiration, transcriptional profiling was performed. Statistically significant changes in mitochondria genes are summarized among the three mutants (Table 3).

Although *hfl1∆* and *rbf1∆* displayed the same reduction in oxygen consumption, mechanistically the decrease in respiration was not identical. For example, the genes that are required for assembly of ETC CIV (*orf19.1691*) and CV (*ATP18*, *orf19.2249*) were down regulated only in *rbf1∆* (Table 3). Other respiratory–related genes such as the ubiquinol cytochrome c reductase (*QCR8* and *QCR9*) were down-regulated in *rbf1*∆ as well. *Candida* species are known to have at least 3 respiratory pathways in addition to the classical ETC chain [18,24]. The other pathways (AOX and PAR) are utilized when the classical pathway is not functioning well. Thus, in *rbf1*∆, an impairment of mitochondrial ETC chain results in subtle increases of *NAD5*, orf19.3353 and a significant increase of *AOX2* (11.5-fold). However, these apparent compensatory changes in the ETC and alternative respiratory route are not sufficient to overcome the mitochondrial dysfunctions in *rbf1*∆. This is especially true for *AOX2*, which is a hallmark gene of a mitochondrial stress response rather than its role in respiration [25].

The transcription profile of genes related to mitochondrial respiration in *hfl1*∆ was different. Instead of a direct impact on ETC complex subunits, we found that the down-regulated genes in this mutant were more related to mitochondrial carbon metabolism such as pyruvate (*ALD6*, *PDK2*), lactate (*DLD1*, *DLD2*) and amino acids (orf19.2184, *ILV6*) (Table 3); also four mitochondrial mtDNA maintenance genes (orf19.3062, *SUV3, CPA1, RAD32*) were transcriptionally reduced in *hfl1*∆. At same time, eight mitochondrial transporters of amino acid/protein, ammonium, FAD, and magnesium are down regulated in *hfl1*∆. Similar to the *rbf1*∆ mutant*,* a possible compensatory response of mitochondrial respiration in *hfl1*∆ was reflected by up-regulation of CI (*NAD6)*, CIV (*COX4*) and others (Table 3). For this same mutant, the only down regulated gene with a respiratory function was *NDE1* (NADH dehydrogenase), which functions as an ETC CI subunit in *S. cerevisiae*.

In contrast, the absence of *DPB4* suppressed expression of *NAD2*, *NAD3*, *NAD4L*, *NAD4*, and *NAD5* more than 2-fold; these five genes comprise the mitochondrial respiration chain CI which are encoded by mitochondrial DNA. Also the mitochondrial nucleotide transporter genes (orf19.28, and *RIM2*) are down regulated in *dpb4Δ* (Table 3). Meanwhile, the house-keeping genes for mtDNA maintenance and mitochondrial RNA processing (a total of 8 genes) were transcriptionally reduced (Table 3). Obviously, Dpb4p regulates respiration in a different manner than the other two TRs.

#### ***Fatty acid catabolism is correlated transcriptionally with decreases in phospholipid (PL) biosynthetic encoding genes***

Similar to mammalian cells, in *C. albicans* lipids provide a source for energy generation via catabolism as well as phospholipid biosynthesis via anabolic pathways [26]. Regulation of both catabolic and anabolic pathways is critical to cell growth [27]. After comparing the transcriptome of lipid metabolism with *goa1*∆, differences are seen among the three TR mutants of *C. albicans*. The absence of *DPB4* resulted in an upregulation of β-oxidation (lipid catabolism) and genes of the peroxisomal glyoxylate cycle (Table 4). But its PL biosynthesis may be compromised since *INO4* (PL biogenesis activator) was down regulated by 100-fold vs. WT cells. The other TRKO strains (*rbf1*∆ and *hfl1*∆) resembled *goa1*∆, and each other, with significant down regulation in lipid oxidation, lipase, the glyoxylate cycle, and peroxisomal importing systems such as the peroxins. In addition, genes for PL biosynthesis including sphingolipid (SL) biosynthesis were down regulated while genes for PL catabolic processes were up regulated. In contrast to the *DPB4* mutant that may regulate PL biosynthetic process, decreased gene expression for lipid catabolism and PL biosynthesis in the other two mutants indicate that *RBF1* and *HFL1* positively regulate both lipid catabolism and PL biosynthesis.

**Table 4:** The transcription profiles of alternative carbon utilization and phenotype-related genes among TRKOs

**Biological processes**	***rbf1Δ***	***hfl1Δ***	***dpb4Δ***
Lipid metabolism	*(n = 62)*^***a***^	*(n = 52)*	*(n = 19)*
	Dw-Peroxins (4/4)^***b***^	Dw-Peroxins (5/5)	
	Dw-lipid catabolism(29/31)	Dw-lipid catabolism(14/17)	**Up**-lipid catabolism (6/9)
	& glyoxylate cycle (2/2)	& glyoxylate cycle(2/2)	& glyoxylate cycle(2/2)
	Dw-PL biosynthesis (10/12)	Dw-PL biosynthesis (15/17)	Dw-PL biosynthesis (4/4)
	**Up**-PL catabolism (3/4)	**Up**-PL catabolism (2/2)	**Up**-PL catabolism (3/3)
	Dw-SL biosynthesis (3/4)	Dw-SL biosynthesis (2/2)	
	Dw-ERG biosynthesis (2/4)	Dw-ERG biosynthesis (3/4)	**Up**-ERG biosynthesis (2/2)
Non-glucose and glucose utilization	*(n = 31)*	*(n = 32)*	*(n = 12)*
	Dw-carbon utilization (26)	Dw-carbon utilization (23)	**Up**-carbon utilization (9)
	*GAL1, GAL10*	*GAL1, 10,102,7*	
	**Up**-fermentation	**Up**-fermentation	**Up**-fermentation
	glycolysis	glycolysis	glycolysis
	glycogen	glycogen	glycogen
	glucose utilization	glucose utilization	glucose utilization
	xylose	trehalose	xylose
Amino acid metabolism	*(n = 31)*	*(n = 28)*	*(n = 19)*
	Dw-aa biosynthesis (8)	Dw-aa biosynthesis (9)	Dw-aa biosynthesis (8)
	**Up**-aa biosynthesis(3)	*MET2,3,10,15*	*MET2,3,6,10,13,14*
	Dw-aa catabolism (5)	Dw-aa catabolism (5)	Dw-aa catabolism (5)
		*ARO3, ARO8*	
	**Up**-aa catabolism(9)	**Up**-aa catabolism(8)	**Up**-aa catabolism (6)
	*ARO9,ARO10*	*ARO9,ARO10*	
	**Up**-sulfur/nitrogen assimilation (6)	Dw-sulfur/nitrogen assimilation (6)	
Morphogenesis	*(n = 27)*	*(n = 33)*	*(n = 17)*
	**Up**-hyphal formation (13)	**Up**-hyphal formation (12)	**Up**-hyphal formation (8)
	*ECE,1 HWP1*,*DEF1, HGC1*,*FGR43*	*ECE1, HWP1*, *FGR18* , *HGC1*	*FGR6-1,3,4,10, RBR1, IHD2*
	*RBR1*, *IHD2,FGR6-1,4,10*	*FGR43, RBR1*,*IHD2*	
Transporters	*(n = 101)*	*(n = 80)*	*(n = 37)*
	Dw: sugar, amino acid, MSF	Dw: sugar, amino acid,MSF	Dw: lactate, polyamine
	sterol/PL, nucleosides,	sterol/PL, nicotinamide,	
	choline, nicotinamide,	CDRs efflux pump, urea	
	ion (K^+^, NH_4_^+^, Ca^+2^, P^-^, Cl^-^)	ion (S^-^, NH_4_^+^, Zn^+2^, P^-^)	
	**Up:** urea, allantoate	**Up:**spermidine/polyamine	**Up:** glucose, acetate, MSF
	spermidine/polyamine	cation (H^+^, Ca^+2^,Cu^+2^, Fe^+3^)	fatty acid, aa,
	cation (H^+^, Cu^+2^, Fe^+3^)		ions (H^+^, Cu^+2^, Fe^+3^ , S^-^)
		

a: Total number of genes in this group;

b: x/y indicates “x” number of genes are down (Dw) or up (Up) regulated among total of “Y” number of genes in this metabolic process.

#### ***Alternative carbon source metabolism is also regulated by each TR***

The biological implications for the assimilation of non-glucose carbon sources even when glucose is not limiting for *C. albicans* has been described [12,28-30]. We observed that numerous genes, required for non-glucose utilization in both *rbf1*∆ (26 of a total of 31 genes) and *hfl1*∆ (23 of 32), were down regulated along with mitochondrial defects. Notably, the *GAL* gene cluster was significantly reduced by 4.6-6.4 fold in *hfl1*∆ (*GAL1, 7, 10, 102*) and 2.9-3.0-fold in *rbf1*∆ (*GAL1, 10*) (Table 4). On the other hand, most of the genes for alternative carbon consumption in *dpb4*∆ increased transcriptionally (9 of 12 in total), including genes for fermentation (*IFD6*), glycogen catabolism, and the xylose catabolic gene *XYL2*. The genes of these three metabolic processes also were upregulated in *RBF1* and *HFL1* mutants. Therefore, we assume that the growth defects of *RBF1* and *HFL1* mutants were also contributed by their reduced ability to use non-glucose carbon sources including lipids mentioned above. However, gene transcription of glycolysis and fermentation was upregulated in each mutant.

#### ***Amino acid metabolism is regulated by each TR***

Regarding genes of amino acid biosynthesis, more genes were downregulated than upregulated for each of the TRKO mutants (Table 4). However, for the *hfl1*∆ and *dpb4*∆, down regulation of methionine synthesis genes were particularly common. Interestingly, transcription of the aromatic amino acid catabolic genes *ARO9* and *ARO10* were up-regulated only in *rbf1*∆ and *hfl1*∆ (Table 4). Both gene products are aromatic transaminases [31]. Their functions are associated with providing an alternative, energy efficient means for NADH regeneration, nitrogen assimilation, and pseudohyphal growth [31]. As stated above, down regulation of the MET genes was observed in *hfl1*∆ and *dpb4∆*. Methionine, as a constituent of proteins, is also critical to biochemical pathways, including the “methyl cycle” which generates the key metabolite S-adnosylmethioinine (AdoMet) [32]. As the main donor of methyl groups in methylation reactions, AdoMet plays a vital role in d*e novo* phosphatidylcholine (PC) synthesis that requires three AdoMet-dependent methylation steps [33].

#### ***Morphogenesis and cell wall responses are regulated by each TF***

The repressive activity of *RBF1* on filamentous growth in *C. albicans* was first noted by Aoki *et al*[22]. In Table 4, we list the most common genes that are related to filamentous growth and their expression level in each mutant. We show that the production of hyphae was associated with the upregulation of genes, such as *RBR1, HWP1* and *ECE1* in *rbf1*Δ and *hfl1*Δ mutants, but much less so in *dpb4*Δ. Transcriptional changes were not noted in the transcription factors *CPH1* and *EFG1*. These partial transcriptional profiles mostly correspond to the hyphal phenotypes of the *rbf1*Δ and *hfl1*Δ mentioned above.

Microarray data support a general increase of genes encoding cell wall β-glucan biosynthesis among three mutants, such as *EXG2*, *PHR1*, *PHR2*, *GSC1* and *KRE1.* Up or down regulation of genes associated with the regulation of mannosylation are noted in the *hfl1Δ* and *rbf1Δ* (Additional file 1: Table S1 and Additional file 2: Table S3). In addition to the cell wall glucan biosynthesis genes, those of the cell wall integrity and MAPK pathways were up-regulated, including the *CHK1* histidine kinase and the *CEK1* MAP kinase. Both genes are known to regulate cell wall polysaccharide synthesis [34,35].

#### ***Regulation of metabolic flux transporters***

The regulatory roles of the three TRs on transporter activity have been noted (Tables 3 and 4). The major changes in both *rbf1*∆ and *hfl1*∆ mutants were down-regulation of transporters for sugar, lipid, amino acids, as well as the MFS transporter family (major facilitating superfamily). Quantitatively, 101 transporters were downregulated in *rbf1*∆, 80 in *hfl1*∆, and 37 in *dpb4*∆, of which the mitochondrial transporters and inter organelle transporters are not included. Certainly, the circuits for nutrient import from extracellular environment or intracellular translocation between compartments are regulated by all TRs but less so by *DPB4*. In *dpb4*∆, gene expression for MFS, sugar, lipid and amino acid importers are increased.

**Figure 7:**
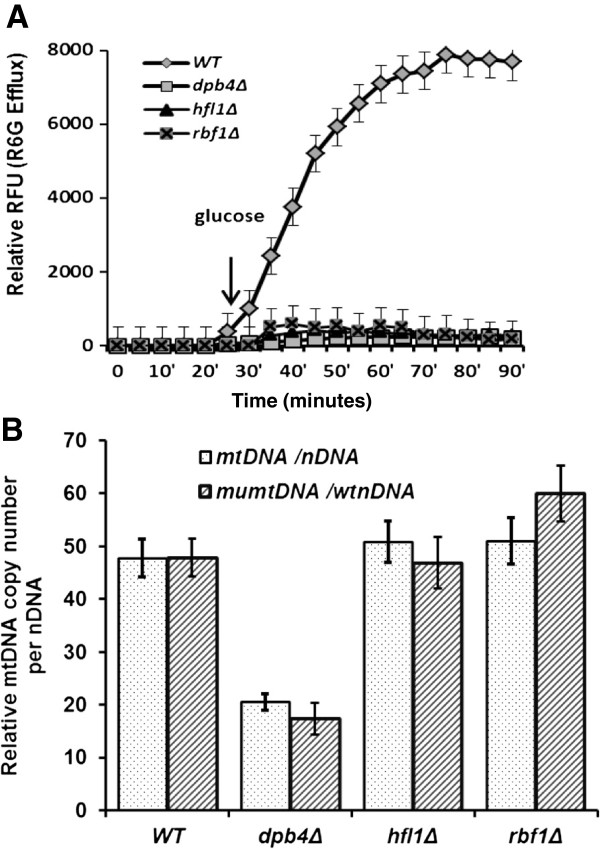
**Membrane transport of R6G is reduced in each TF mutant and relative mtDNA copy number is less in *dpb4*∆ mutant (7B). (A)** Parental (SN250) and each mutant were assayed for transport of R6G. Relative RFU, relative fluorescent units over a 90 min time interval were determined. Cells were starved in buffer for 20 min then glucose was added to each culture. Compared to parental cells, all mutants had little transporter activity. **(B)** The ratio of mtDNA copy number to that of nuclear DNA (nDNA) is calculated by ΔC_t_ with matched pairs of mtDNA/nDNA primers. The relative copy number of mtDNA is averaged from three biological replicates (mtDNA/nDNA). Compared to the parental strain, *dpb4*Δ has less mtDNA copies compared to its own nDNA or parental nDNA. However mutants (*rbf1Δ* and *hfl1Δ*) have a similar mtDNA copy number as the parental strain*.* (mumtDNA: mutant mtDNA; wtnDNA: wild type strain nDNA).

The measurement of intracellular accumulation of R6G is a useful method to reflect the activity of the CDR drug efflux pumps. The extracellular release of R6G in *C. albicans* was inversely correlated with the level of this group of efflux exporters [36]. Similar to *goa1*∆, the CDR genes (*CDR2*, *CDR4* and *CDR11*) are down regulated in *hfl1*∆, which may explain its poor extracellular efflux rate of R6G shown in Figure 7 and hypersusceptibility to fluconazole (Table 2). However, these CDR genes were not changed in *rbf1*∆ and *dpb4*∆ although they displayed a similar rate of R6G efflux as *hfl1∆*. Rbf1p, Hfl1p, or Dpb4 may regulate efflux by a different mechanism. Because R6G has a permanent positive charge, its cellular accumulation relies on a plasma membrane potential that is localized mainly in the mitochondria [37].

The spermidine transporter was only upregulated in *rbf1*∆ and *hfl1Δ*. These data may illustrate that these mutants have a high demand for sustaining intracellular pH and membrane potential since the spermidine transporter synchronizes Ca^2+^, Na^+^, K^+^ -ATPase in plant cells [38]. However, transporters of metal cations were upregulated in each of the TRKO mutants. The significance of uptake of Fe^3+^ and Cu^2+^ uptake is related to mitochondrial respiration since electron transfer among ETC complexes is carried out by reduced metal ions [39]. The high demand for metal ion uptake in the TRKO mutants again suggests their defective mitochondria.

#### ***Dpb4p is required for mitochondrial genome maintenance in C. albicans***

Giving the fact that a few complex I genes are down-regulated transcriptionally in *dpb4Δ* described above (Figure 4), we performed real-time PCRs to determine if mtDNA maintenance is affected in this mutant with four sets of primers: two sets of mtDNA encoded genes NAD1 (complex I subunit) and COX1 (complex IV subunit), and two sets of nDNA genes (18S rRNA and SOD1). The average number of copies of mtDNA per nDNA for DPB4 TRKO strain is less than half the levels of WT and other two mutants tested in Figure 7B. Since nDNA replication is also extensively affected in *dpb4Δ* mutant microarray data, we also normalized the mtDNA copy number by comparing the mtDNA Ct of *dpb4Δ* with nDNA Ct of WT cells. Again we see the reduction of mtDNA replication rate in this mutant.

#### ***The TRs regulate other TRs***

**Figure 8:**
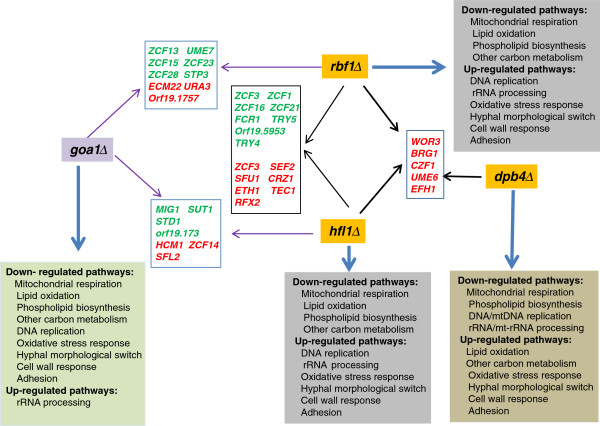
**TRKOs and their influence on transcription of other TR genes.** Each TRKO mutant and *goa1*∆ is shown with arrows that connect common TR genes (rectangular boxes) affected in each null mutant. The TR genes are indicated in red (upregulated) or green (down regulated). Thus for *dpb4*∆, 5 upregulated genes are shown (connected by a right-facing black arrow) that are common to both *rbf1*∆ and *hfl1*∆. Common TR genes of *rbf1*∆ and *hfl1*∆ are similarly inked by black arrows. The number of TR genes common to both *rbf1*∆ and *hfl1*∆ is much greater than those common to *dpb4*∆. Also shown are comparisons of presumed up or down regulated pathways for each mutant including *goa1*∆ (square boxes of various colors).

In eukaryotes, ~3-5% of their protein repertoire is transcription factors [40]. In our previous studies of *GOA1*, we found ~100 transcription factors that were down regulated in the gene-deleted mutant, including reduction of a large group of Zn2-Cys6 cluster TRs. Presumably, the TRs regulate metabolic pathways. However, of 77 such genes, most are poorly characterized, yet they are fungal-specific [41]. We compared the regulation of other TRs by *RBF1*, *HFL1* and *DPB4*. The TRs regulated by *RBF1* and *HFL1* are closely related. Fifteen of the TR genes were either up or down regulated (Figure 8 and see Discussion). In this group, some genes shared between *RBF1* and *HFL1* mutants were also changed in the *GOA1* mutant, such as *ZCF1, ZCF5, ZCF16, ZCF21, FCR1, TRY4* and *RFX2.* The likely involvement of these 7 TRs in the regulation of metabolic process needs to be determined, although the last three transcriptional regulators have been reported to process filamentous growth and azole drug resistance [42,43].

Only 5 common gene changes were noted in the three TRKOs (Figure 8). Interestingly, the predicated functions of *UME6, WOR3, BRG1, CZF1* and *EFH1* are all associated with hyphal formation or the white-opaque switch that actually matches their hyphal phenotypes. However, these genes were down-regulated in *goa1Δ* which correlates with the inability of that strain to form hyphae under hyphal inducing conditions. In conclusion, *RBF1*, *HFL1* and *DPB4* are repressors of the five genes mentioned above for morphologic switching. Overall, the regulatory network for *DPB4* mutant is very different, since only 14 of TRs were changed in *dpb4Δ* compared to 44 for *rbf1Δ* and 31 for *hfl1Δ,* including the five common TRs just mentioned above and two other TRs all of which relate to morphogenesis.

## Discussion

Goa1p is required (directly or indirectly) for a number of cell processes [16-20]. Those processes include homeostasis of stationary phase cells, morphogenesis, prevention of early apoptosis, ATP production through its regulation of the ETC CI, and communication between peroxisomes and mitochondria. The latter function ensures that both organelles coordinate pathways of energy production, including aerobic respiration, β-oxidation, gluconeogenesis, and carbon conservation via the glyoxylate cycle. We believe these are the core functions of Goa1p. Deletion of *GOA1*, achieved by constructing a null mutant, causes loss of chronological aging, constitutive yeast growth, lethal levels of ROS, apoptosis, heightened susceptibility to triazole antifungal drugs, and reduced energy production from both mitochondria and peroxisomes. The host-pathogen outcomes from a lack of Goa1p are avirulence, a failure to colonize kidney tissues, and increased killing by human neutrophils and macrophages [16,20].

The availability of a transcription regulator knockout (TRKO) library proved suitable for facile screening based upon the ability of each mutant to grow or not on YP medium containing glycerol as the sole carbon source. Six such mutants were then reduced to three whose gene absence caused a down regulation of *GOA1* indicating that each TR is a positive regulator of *GOA1*. However, we have not as yet identified gene targets of each TF. Thus, our supposition of direct or indirect regulation by each TF awaits confirmation. Our ongoing studies focus on chromatin immunoprecipitation (ChIP) to identify gene targets of each TF.

We have not completed phenotyping profiles of the TRKOs except for antifungal susceptibilities and respiratory competency. In this regard, Rbf1p and Hfl1p are similar to Goa1p in that they each are required for maintenance of WT levels of fluconazole susceptibility. Each of the three TRs is required for respiratory activities but to varying degrees. For example, Rbf1p is much more critical to the maintenance of CI activity and, consequently, low levels of ROS. Each of the TRs is a negative regulator of morphogenesis (yeast-hyphal transition), yet Goa1p would appear to be part of a positive regulatory circuit. Further, Goa1p is critical to host cell recognition. Loss of *GOA1* results in a downregulation of genes encoding this function (17-19). Oppositely, the TR mutants display upregulation of these genes. This difference needs to be correlated with the binding of the TRKOs to epithelial and innate immune cells. The cell wall inhibitor studies may point to a role of each TF in compensatory changes in response to cell wall defects caused by inhibitors. A last look at functional annotation is that of transporter activity, of which each of the TRs and Goa1p is essential (Figure 7A).

An important advantage of the TRKO mutants is to identify genes that are common to specific biological processes. For example, *SUV3* is the only common down-regulated gene of mitochondrial genome maintenance for all TR mutants (Table 3). *SUV3* is an RNA helicase that is essential for mitochondrial RNA catabolism and respiratory growth. Oppositely, for the filamentous morphogenesis circuitry, three genes among several are commonly upregulated in each TR [*RBR1* and IHD2 (Table 4)].

To visualize the overall roles of each TR and Goa1p, we aligned genes that were up or down regulated in each mutant and also shared among the TRKOs. Also, for each TR, we have summarized the gene responses as presumed functional activities (Figure 8). The three TRs share similar roles in metabolic pathways, stress responses, cell wall regulation, fluconazole susceptibility, and morphogenesis, but transcriptional changes are different. As stated previously, there was a higher number of genes committed to metabolic processes in Rbf1p and Hfl1p than in Dbp4p regarding alternative carbon utilization of non-favorable carbon, lipid and amino acid catabolism. Oppositely, metabolic regulation performed by Dbp4p is likely because of its role in the maintenance of mtDNA genome (Figure 7B). Thus, when mtDNA replication rate in *dpb4Δ* is compromised, the cell metabolism and respiration are also then affected. While Rbf1p and Hfl1p regulated lipid oxidation and other peroxisomal functions such as the glyoxylate pathway, Dbp4p is less critical to these processes. Likely, phospholipid homeostasis in *C. albicans* is also contributed by each TR but in different ways. In fact, a more than 100-fold reduction of *INO4* suggests that Dpb4p provides a positive regulation of PL biosynthesis. Ino4p is the PL biosynthetic activator that forms a heterodimer with Ino2p to regulate UAS_INO_-containing genes [44]. When PA levels drop in ER, the Opi1p repressor disassociates from the nuclear/ER membrane, then translocates to nuclei to attenuate the Ino2p-Ino4p complex activity [45].

Oxidative stress caused by ROS is usually associated with aberrations of mitochondrial metabolism that can threaten cell survival [46]. In this regard, the levels of cell ROS are quite different among the TRKO mutants. ROS production by *rbf1*∆ was much higher than in *hfl1*∆, while that of *dpb4*∆ was similar to WT cells. However, most stress response genes in *rbf1*∆ and *hfl1*∆ were similarly represented. Perhaps the higher level of ROS in *rbf1*∆ is due to the combined loss of CI and CIV activities. CI accounts for ~ 50% of the oxidoreduction activity of the ETC, and if dysfunctional, one could expect much more ROS as superoxide. Another major functional difference in the three TRs as compared to Goa1p is that they negatively regulate genes associated with cell wall and cell integrity while Goa1p positively regulates this gene cluster including the signaling transduction pathway MAPK *CEK1* and *CHK1*[24,25]. Again, direct versus indirect regulatory effects need to be sorted out. Also, as stated above, Goa1p is required for the yeast to hypha phase transition along with cell surface GPI-anchored adhesins, while each of the three TRs repress these growth requirements. Finally, we note again that Dbp4p positively regulates nuclear DNA/mtDNA replication or maintenance as well as rRNA/mt-rRNA processing. The assignment of genes in these groups quantitatively is much greater in Dbp4p than the other two TRs.

Translational applications of these data are apparent. The TFs are fungal-specific, and therefore are of reasonable purpose as drug targets for antifungal drug discovery. Inhibitors likely will have severe consequences on cell metabolism and growth. High throughput screens for such inhibitors might utilize heterozygote libraries of TRs. Hypersensitivity of strains to compounds compared to a parental strain indicates a haploinsufficiency response (decreased fitness) suggesting a drug target. Or, screens of the TRKO mutants with a compound library may be useful. In this case, a resistance phenotype likely indicates the target of the compound.

## Conclusions

Our data are the first to integrate the regulation of metabolic processes by the transcription regulators Rbf1p, Hfl1p and Dpb4p. That these TRs regulate mitochondria and peroxisomal metabolism also is new to fungal pathogens and speaks to their regulation of organelle cross-talking. Equally important, each TR was previously unstudied except for two reports on Rbf1p and its role as a negative regulator of morphogenesis in *C. albicans*. Of particular interest is that there is little overlap in genes of mitochondrial functions regulated by the 3 TRs, yet each is functionally important to this organelle (Table 3). In regard to other cell metabolic activities, such as lipid metabolism, non-glucose utilization, and amino acid metabolism, Rbf1p and Hfl1p are more similar in their gene profiles compared to Dpb4p (Table 4). Two of the major differences in gene transcription among the TRs are transporter genes, of which Rbf1p and Hfl1p clearly are of broader scope than Dpb4p. Second, Dpb4p is a regulator for mtDNA genome maintenance. These observations provide the beginning of an integrative view of global cell functions by transcriptional regulators of fungi.

## Methods

### Strains and media

The homozygous mutants (TRKO) of a *C. albicans* transcription factor *rbf1∆*, *hfl1∆* and *dpb4∆* (orf19.2088) were obtained from transcription factor (TR) library provided by Dr. Alexander Johnson’s lab [21]. All assays with these mutants include the parental strain *C. albicans* SN250 [21]. All strains were grown at 30°C in 2% YPD, 2% glycerol YPG medium (glycerol used as carbon source), minimal medium (0.67% yeast nitrogen base) containing 2% glucose (SD), or Spider agar plates and YPD at pH 9.5.

### TR deletion library screens

The entire TRKO mutant library was initially screened in parallel YPD and YPG plates. For these experiments, TR mutants with mitochondrial defects were identified by their lack of growth on YP-glycerol. 3 μl of cells was added to YPD or YP-2% glycerol (YPG) plates. Plates were kept at 30°C incubator for 24 hours and those with growth defects in YPG only were identified and verified by a second screening with a second deletion strain of the same gene. Subsequent experiments were done only with those mutants that had defective growth in YPG.

### Morphology and generation times

All strains were grown overnight in YPD at 30°C for all experiments described below. Cells were washed, diluted to a cell suspension of 1 × 10^5^/ul, and streaked on YPD, pH 9.5 or Spider agar media and incubated at 30°C. Plates were observed on day 7 and photographed. The morphologic switch from yeast to filamentous forms in 10% serum at 37°C for all strains was done with the same growth conditions. Mutants were compared to SN250. Generation times for *rbf1*∆, *hfl1*∆*,* and *dpb4*∆ strains were evaluated as described [16]. All strains were grown in YPD media at 30°C for 20 hours and cell suspensions were adjusted to an initial cell concentration of OD = 0.1. Also, since the mutants were constitutively filamentous, 50 ml of each culture was centrifuged, and cell pellets were dried, and weighed every 2 hours. Doubling time was determined based on the biomass for each strain in duplicate cultures.

### Functional mitochondrial assays

The measurement of oxygen consumption, reactive oxidant species (ROS) levels, and mitochondrial enzymatic activities of each mutant (*rbf1*, *hfl1* and *dpb4*) and SN250 were done as described [21]. In brief, for oxygen consumption experiments, each strain was inoculated into 100 ml of YPD (2% glucose) broth until exponential growth was achieved. Cells were washed twice with PBS and suspended into fresh YPD at a cell concentration of OD = 0.3. 1 ml of cells was then loaded immediately into the sealed respirometer chamber (Hansatech Instruments Ltd., Norfolk, England). Oxygen consumption was measured over 10 min and polarographically recorded using Oxygraph Plus software. The remaining cultures were centrifuged to determine cell biomass. Oxygen consumption is presented as nmol per min per mg cell dry weight. Data from three experiments were averaged.

Intracellular ROS levels for each strain were evaluated by staining cells using the ROS sensitive fluorescent dye DCFDA (2′,7′-dichlorofluorescein diacetate; Sigma). Since growth was filamentous, the final step in ROS measurement was performed using a fluorescence microplate reader in 96-well black plates (Dynex Technologies Inc., Chantilly, VA, USA) at λex: 485 nm and λem: 530 nm. Cell suspensions were kept in the dark to minimize loss of fluorescent signal during the assay. Cell cultures for each strain were prepared in 20 ml of YPD using an inoculum of 5 × 10^4^/ml; cells were grown overnight at 30°C, in shake culture (200 rpm). The cell pellets from 1 ml of cultures were washed once with PBS and suspended to 1 ml of PBS with 50 μM DCFDA for 30 min at 30°C, 100 rpm. Cells were washed twice with PBS, and 200 μl from each strain was introduced into a 96-well microtiter plate. Cell fluorescence in the absence of DCFDA was used to verify that background fluorescence was similar per strain. ROS data was obtained from duplicate cultures, and all experiments were repeated a total of 3 times.

Enzyme activities of the mitochondrial electron transport chain (ETC) CI and CIV were measured spectrophotometrically following procedures described previously [17,18]. CI (NADH:ubiquinone oxidoreductase) and CIV (cytochrome c oxidase) activities are plotted from duplicated samples for each strain as nmol per min per μg of mitochondrial protein.

### Antifungal susceptibility tests

The susceptibility (MIC_50_ and MIC_100_) for all strains to fluconazole, amphotericin B (AmB) and caspofungin was determined using the broth microdilution method according to CLSI guidelines M27-A3. The range of drugs tested was 0.25-256 μg/ml for fluconazole; 0.03-32 μg/ml for AmB; and 0.016-16 μg/ml for caspofungin. Exponentially grown cultures for each tested strain were diluted in RPMI-1640 to a density of 1 × 10^4^ CFU/ml and 100 μl was added to each well of 96-well plate containing 100 μl RPMI-1640 with different concentration of drug. All plates were incubated for 48 h at 37°C. The MIC_100_ was determined as the concentration resulting in complete growth inhibition, and MIC_50_ for fluconazole corresponded as an inhibition of at least 50% of fungal growth.

### Cell wall and ETC CI and CIV inhibitor assays

Overnight cultures of all strains were collected and washed twice with PBS. The cell suspension, adjusted to 5 × 10^5^ to 5 × 10^1^ in 10 μl PBS, was spotted onto YPD agar with or without inhibitors. For identifying the cell wall defects, 25 μg/ml of calcofluor white (CFW) or Congo red (CR) was added to YPD plates. CI and CIV inhibitors were used at concentrations of 10 μM rotenone and 10 mM KCN in YPD agar. Cultures were incubated at 30°C for 24 h and photographed.

### Rhodamine 6G (R6G) efflux

These experiments were performed using a modified procedure of our earlier published data [19] using 96-well microtiter plates. In brief, cells were initially seeded into 10 ml of fresh YPD after an overnight culture. Exponentially growing cells were washed twice with PBS (pH 7.0, without glucose), and suspended in glucose-free PBS to 10^8^/ml for 2 hours incubation to deplete glucose. Rhodamine 6G was then added at a final concentration of 10 μM for 20 min. Again, cells were washed and suspended in glucose-free PBS before introducing 2% glucose. At every 10 min base, 0.2 ml of cells were removed and energy-dependent efflux of R6G was measured by monitoring the absorption at 527 nm in that were transferred into a black 96-well plate in triplicate, glucose-free controls were included in all experiment.

### Quantitative PCR analysis of Mitochondrial DNA (mtDNA) replication rate

The total DNAs were isolated from SN250 strain and mutants using RNase to remove RNA followed by standard phenol/chloroform extraction and ethanol precipitation. The concentration of DNAs was determined by a nano-spectrophotometer. The primers for analysis of mtDNA are NAD1F (5′-TAGGTTGTGTTGCTGAATGTGC) and NAD1R (5′-CCAGTACCACCACCCATAAATAAG), COX1F (5′-GGTGAATTACGTCTAGCTGTTCC) and COX1R (GCACCATCTAATAGCCCTACTCA). Two sets of nuclear DNA (nDNA) gene are 18SrRNAF (5′-CGCAAGGCTGAAACTTAAAGG) and 18SrRNAR (5′-AGCAGACAAATCACTCCACC), SOD1F(5′-GCTCCAACCACAATTTCCTG) and SOD1R (5′-TGGATTGAAATGAGGACCAGC). The 20 μL PCR reaction contains 1× iQSyBR green supermix (Bio-Rad), 0.25 μM of each primer, and approximately 5 ng of total genomic DNA for each strain. PCR conditions are 2 min at 95°C, followed by 40 cycles of 15 s of denaturation at 95°C and 30 s of annealing at 55°C and 30s of extension at 60°C. The relative copy number of mitochondrial DNA over the nuclear DNA was averaged from the threshold cycle number (C_t_) difference for each pairs of mtDNA/nDNA [47,48]. The individual ratio was determined from each sets of mtDNA/nDNA pairs use the calculation equation N = 2^ΔCt^ where ΔC_t_ = Ct^nDNA1^ -Ct^mDNA1^ or ΔC_t_ = Ct^nDNA2^ -Ct^mDNA2^. Statistical analysis of data was conducted by the *t* test.

### RNA and microarray analyses

For transcriptional profiling, RNA was obtained from the TRKO mutants and SN250 grown in 20-ml of 2% SD medium at 30°C for 5 h as previously described [17,19]. RNA was quantified using an RNA 6000 Nano device, and RNA integrity was assessed using an Agilent 2100 bioanalyzer. For real time PCR measurement of *GOA1* and *NDH51* transcription, overnight cultures in YPD were seeded into 20 ml of fresh SD medium containing 2% glucose. When exponential growth was achieved for all strains, cells were collected and washed, then suspended in YPG medium for one hour before RNA was extracted. Approximately 800 ng of RNA was used to prepare cDNA. Quantitative real-time PCR was carried out in 20 μl of 1x iQ SyBR green Supermix (Bio-Rad) containing 0.25 μM concentration of each primer. The experiment was performed in triplicate using Bio-Rad iQ5, and the transcription level of each gene was normalized to *C. albicans* 18S rRNA levels. The 2^–ΔΔ^CT method of analysis was used to determine the fold change in gene transcription [17,18].

One-color microarray-based gene expression analysis was done using the Agilent low input Quick Amp Labing kit. The RNAs for each strain were prepared from exponential cells cultured in 20-ml of SC medium containing 2% glucose. cDNA was synthesized from 100 ng total RNA for each strain according to the manufacturer’s instructions. Hybridization was completed in a Agilent SureHyb hybridization chamber and scan processed with an Agilent SCAN G2505C Microarray Scanner System. The array used in this study was provided by Agilent Technologies (eArray, ID 037557). The total of 6101 genes (including 12 mitochondrial genes) was done in duplicate.

The image files were first analyzed by Agilent Feature Extraction Software and cyanine 3 intensities were then logarithmically transformed and statistically normalized. The fold change for each gene was calculated by comparing to wild type. In this study, we adopted the cut off for the parametric *p*-value <0.05 and fold change >2 to determine the significance. The entire significant genes list for *rbf1Δ*, *hfl1Δ* and *dpb4Δ* are available in the supplemental material (Additional file 1: Table S1, Additional file 3: Table S2 and Additional file 2: Table S3).

### Availability of supporting data

The microarray data of three TRKO strains and wild type SN250 have been deposited to the GEO database with accession number [GEO:GSE54057]. The microarray data of each mutant with gene changes more than 2- fold are included in this manuscript as additional files indicated below.

## Abbreviations

AA: Amino acid; ALS: Agglutinin-like sequence; CFW: Calcofluor white; CLSI: The Clinical and Laboratory Standards Institute; CR: Congo red; ERG: Ergosterol; ETC: Electron transport chain; MIC: Minimum inhibitory concentration; PA: Phosphatidic acid; PL: Phospholipid; R6G: Rhodamine 6G; ROS: Reactive oxidant species; SL: Sphingolipid; TR: Transcription regulator; TRKO: Transcriptional regulator knockout library; UASINO: Upstream activation sequence *INO*; WT: Wild type.

## Competing interests

The authors declare no competing interests exist.

## Authors’ contributions

P.S performed the TRKO library screening and partial functional study; K.K and D.L performed the most of functional studies, morphological studies, Q-PCR and microarray data analysis; N.S and D.L performed microarray assay; R.C and D. L provided the theoretical framework and guidance for this study and wrote the manuscript. All authors read and approved the final manuscripts.

## Supplementary Material

Additional file 1: Table S1 Up and down-regulated genes list in *rbf1Δ* mutant. Click here for file

Additional file 2: Table S3 Up and down-regulated genes list in *dpb41Δ* mutant. Click here for file

Additional file 3: Table S2 Up and down-regulated genes list in *hfl1Δ* mutant. Click here for file

## References

[B1] Brown GD Denning DW Gow NA Levitz SM Netea MG White TC Hidden killers: human fungal infections Sci Transl Med 2012 15 165rv13 23253612 10.1126/scitranslmed.3004404

[B2] Calderone R Gay-Andrieu F Li D Alex D Sun N Tegos G, Mylonakis E Antifungals and antifungal discovery, chapter 17 Antimicrobial Drug Discovery 2012 UK: CABI

[B3] Arnold HM Micek ST Shorr AF Zilberberg MD Labelle AJ Kothari S Kollef MH Hospital resource utilization and costs of inappropriate treatment of candidemia Pharmacotherapy 2010 15 361 368 10.1592/phco.30.4.361 20334456

[B4] Gauwerky K Borelli C Korting HC Targeting virulence. A new paradigm for antifungals Drug Discov Today 2009 15 214 222 10.1016/j.drudis.2008.11.013 19152839

[B5] Pfaller M Neofytos D Diekema D Azie N Meier-Kriesche HU Quan SP Horn D Epidemiology and outcomes of candidemia in 3648 patients: data from the Prospective Antifungal Therapy (PATH Alliance^®^) registry, 2004-2008 Diagn Microbiol Infect Dis 2012 15 323 31 10.1016/j.diagmicrobio.2012.10.003 23102556

[B6] Blumberg HM Jarvis WR Soucie JM Edwards JE Patterson JE Pfaller MA Rangel-Frausto MS Rinaldi MG Saiman L Wiblin RT Wenzel RP National Epidemiology of Mycoses Survey(NEMIS) Study Group Risk factors for *Candida* blood stream infections in surgical intensive care unit patients Clin Infect Dis 2001 15 177 186 10.1086/321811 11418877

[B7] Thompson GR 3rd Patel PK Kirkpatrick WR Westbrook SD Berg D Erlandsen J Redding SW Patterson TF Oropharyngeal candidiasis in the era of anti-retroviral therapy Oral Surg Oral Med Oral Pathol Oral Radiol Endo 2010 15 488 495 10.1016/j.tripleo.2009.11.026 PMC2843789 20156694

[B8] Gagne J Goldfarb N Candidemia in the in-patient setting: treatment options and economics Expert Opin Pharmacother 2007 15 1643 1650 10.1517/14656566.8.11.1643 17685882

[B9] Grumaz C Lorenz S Stevens P Lindemann E Schöck U Retey J Rupp S Sohn K *Species and condition specific adaptation of the transcriptional landscapes in* Candida albicans *and* Candida dubliniensis BMC Genomics 2013 15 212 10.1186/1471-2164-14-212 23547856 PMC3626586

[B10] Shahi P Moye-Rowley WS Coordinate control of lipid composition and drug transport activities is required for normal multidrug resistance in fungi Biochim Biophys Acta 2009 15 852 859 10.1016/j.bbapap.2008.12.012 19150512 PMC2671576

[B11] Sandai D Yin Z Selway L Stead D Walker J Leach MD Bohovych I Ene IV Kastora S Budge S Munro CA Odds FC Gow NA Brown AJ The evolutionary rewiring of ubiquitination targets has reprogrammed the regulation of carbon assimilation in the pathogenic yeast *Candida albicans* MBio 2012 15 e00495-12 doi: 10.1128 23232717 10.1128/mBio.00495-12 PMC3520108

[B12] Niimi M Kamiyama A Tokunaga M Respiration of medically important *Candida* species and *Saccharomyces cerevisiae* in relation to glucose effect J Med Vet Mycol 1988 15 195 198 10.1080/02681218880000271 3050010

[B13] Ramírez MA Lorenz MC Mutations in alternative carbon utilization pathways in *Candida albicans* attenuate virulence and confer pleiotropic phenotypes Eukaryot Cell 2007 15 280 290 10.1128/EC.00372-06 17158734 PMC1797957

[B14] Barelle CJ Priest CL Maccallum DM Gow NA Odds FC Brown AJ Niche-specific regulation of central metabolic pathways in a fungal pathogen Cell Microbiol 2006 15 961 971 10.1111/j.1462-5822.2005.00676.x 16681837 PMC1472618

[B15] Lorenz M Bender J Fink G Transcriptional response of *Candida albicans* upon internalization by macrophages Eukaryot Cell 2004 15 1076 1087 10.1128/EC.3.5.1076-1087.2004 15470236 PMC522606

[B16] Bambach A Fernandes MP Ghosh A Kruppa M Alex D Li D Fonzi WA Chauhan N Sun N Agrellos OA Vercesi AE Rolfes RJ Calderone R Goa1p of *Candida albica*ns localizes to the mitochondria during stress and is required for mitochondrial function and virulence Eukaryot Cell 2009 15 1706 1720 10.1128/EC.00066-09 19717740 PMC2772395

[B17] Chen H Calderone R Sun N Wang Y Li D Caloric restriction restores the chronological life span of the Goa1 null mutant of *Candida albicans* in spite of high cell levels of ROS Fungal Genet Biol 2012 15 1023 32 10.1016/j.fgb.2012.09.007 23063955

[B18] Li D Chen H Florentino A Alex D Sikorski P Fonzi WA Calderone R Enzymatic dysfunction of mitochondrial complex I of the *Candida albicans goa1* mutant is associated with increased reactive oxidants and cell death Eukaryot Cell 2011 15 672 682 10.1128/EC.00303-10 21398508 PMC3127659

[B19] Sun N Fonzi W Chen H She X Zhang L Zhang L Calderone R Azole susceptibility and transcriptome profiling in *Candida albicans* mitochondrial electron transport chain complex I mutants Antimicrob Agents Chemother 2013 15 532 542 10.1128/AAC.01520-12 23147730 PMC3535965

[B20] She X Zhang L Chen H Calderone R Li D Cell surface changes in the *Candida albicans* mitochondrial mutant goa1Δ are associated with reduced recognition by innate immune cells Cell Microbiol 2013 15 doi:10.1111/cmi.12135 10.1111/cmi.12135 PMC3738057 23490206

[B21] Homann OR Dea J Noble SM Johnson AD A phenotypic profile of the *Candida albicans* regulatory network PLoS Genet 2009 15 e1000783 10.1371/journal.pgen.1000783 20041210 PMC2790342

[B22] Aoki Y Ishii N Watanabe M Yoshihara F Arisawa M Rbf1 (RPG-box binding factor), a transcription factor involved in yeast-hyphal transition of *Candida albicans* Nippon Ishinkin Gakkai Zasshi 1998 15 67 71 10.3314/jjmm.39.67 9580030

[B23] Levitin A Whiteway M The effect of prostaglandin E2 on transcriptional responses of *Candida albicans* Microbiol Res 2007 15 201 210 10.1016/j.micres.2007.02.001 17433645

[B24] Chamilos G Lewis RE Kontoyiannis DP Inhibition of *Candida parapsilosis* mitochondrial respiratory pathways enhances susceptibility to caspofungin Antimicrob Agents Chemother 2006 15 744 747 10.1128/AAC.50.2.744-747.2006 16436735 PMC1366909

[B25] Joseph-Horne T Hollomon DW Wood PM Fungal respiration: a fusion of standard and alternative components Biochim Biophys Acta 2001 15 179 195 10.1016/S0005-2728(00)00251-6 11245784

[B26] Nohturfft A Zhang SC Coordination of lipid metabolism in membrane biogenesis Annu Rev Cell Dev Biol 2009 15 539 566 10.1146/annurev.cellbio.24.110707.175344 19575637

[B27] Shingu-Vazquez M Traven A Mitochondria and fungal pathogenesis: drug tolerance, virulence, and potential for antifungal therapy Eukaryot Cell 2011 15 1376 1383 10.1128/EC.05184-11 21926328 PMC3209048

[B28] Zhou H Lorenz M Carnitine acetyltransferases are required for growth on non-fermentable carbon sources but not for pathogenesis in *Candida albicans* Microbiology 2008 15 500 509 10.1099/mic.0.2007/014555-0 18227254

[B29] Carman A Vylkova S Lorenz M Role of acetyl coenzyme A synthesis and breakdown in alternative carbon source utilization in *Candida albicans* Eukaryot Cell 2008 15 1733 1741 10.1128/EC.00253-08 18689527 PMC2568070

[B30] Lorenz M Fink G The glyoxylate cycle is required for fungal virulence Nature 2001 15 83 86 10.1038/35083594 11452311

[B31] Hazelwood LA Daran JM van Maris AJ Pronk JT Dickinson JR The ehulich pathway for fusel alcohol production: a century of research on *Saccharomyces cerevisiae* metabolism Appl Environ Microbiol 2008 15 2259 2266 10.1128/AEM.02625-07 18281432 PMC2293160

[B32] Barz T Ackermann K Pyerin W Control of methionine biosynthesis genes by protein kinase CK2-mediated phosphorylation of Cdc34 Cell Mol Life Sci 2006 15 2183 2190 10.1007/s00018-006-6213-5 16952051 PMC11136312

[B33] Malanovic N Streith I Wolinski H Rechberger G Kohlwein SD Tehlivets O S-adenosyl-L-homocysteine hydrolase, key enzyme of methylation metabolism, regulates phosphatidylcholine synthesis and triacylglycerol homeostasis in yeast: implications for homocysteine as a risk factor of atherosclerosis J Biol Chem 2008 15 23989 2399 10.1074/jbc.M800830200 18591246 PMC3259781

[B34] Eisman B Alonso-Monge R Román E Arana D Nombela C Pla J The Cek1 and Hog1 mitogen-activated protein kinases play complementary roles in cell wall biogenesis and chlamydospore formation in the fungal pathogen *Candida albicans* Eukaryot Cell 2006 15 347 358 16467475 10.1128/EC.5.2.347-358.2006 PMC1405885

[B35] Li D Williams D Lowman D Monteiro MA Tan X Kruppa M Fonzi W Roman E Pla J Calderone R The *Candida albicans* histidine kinase Chk1p: signaling and cell wall mannan Fungal Genet Biol 2009 15 731 41 10.1016/j.fgb.2009.06.008 19563901 PMC2731578

[B36] Maesaki S Marichal P Vanden Bossche H Sanglard D Kohno S Rhodamine 6G efflux for the detection of CDR1-overexpressing azole-resistant *Candida albicans* strains J Antimicrob Chemother 1999 15 27 31 10.1093/jac/44.1.27 10459807

[B37] Saengkhae C Loetchutinat C Garnier-Suillerot A Kinetic analysis of rhodamines efflux mediated by the multidrug resistance protein (MRP1) Biophys J 2003 15 2006 2014 10.1016/S0006-3495(03)74628-1 12944313 PMC1303372

[B38] Zhao F Song CP He J Zhu H Polyamines improve K^+^/Na^+^ homeostasis in barley seedlings by regulating root Ion channel activities Plant Physiol 2007 15 1061 1072 10.1104/pp.107.105882 17905858 PMC2048800

[B39] Brandt ME Vickery LE Charge pair interactions stabilizing ferredoxin-ferredoxin reductase complexes. Identification by complementary site-specific mutations J Biol Chem 1993 15 17126 30 8349601

[B40] van Steensel B Mapping of genetic and epigenetic regulatory networks using microarrays Nat Genet 2005 15 Suppl S18 24 15920525 10.1038/ng1559

[B41] Vandeputte P Ischer F Sanglard D Coste AT In vivo systematic analysis of *Candida albicans* Zn2-Cys6 transcription factors mutants for mice organ colonization PLoS ONE 2011 15 e26962 10.1371/journal.pone.0026962 22073120 PMC3205040

[B42] Hao B Clancy CJ Cheng S Raman SB Iczkowski KA Nguyen MH *Candida albicans* RFX2 encodes a DNA binding protein involved in DNA damage responses, morphogenesis, and virulence Eukaryot Cell 2009 15 627 639 10.1128/EC.00246-08 19252121 PMC2669197

[B43] Talibi D Raymond M Isolation of a putative *Candida albicans* transcriptional regulator involved in pleiotropic drug resistance by functional complementation of a pdr1 pdr3 mutation in *Saccharomyces cerevisiae* J Bacteriol 1999 15 231 240 9864335 10.1128/jb.181.1.231-240.1999 PMC103554

[B44] Kumme J Dietz M Wagner C Schüller HJ Dimerization of yeast transcription factors Ino2 and Ino4 is regulated by precursors of phospholipid biosynthesis mediated by Opi1 repressor Curr Genet 2008 15 35 45 10.1007/s00294-008-0197-7 18542964

[B45] Henry SA Kohlwein SD Carman GM Metabolism and regulation of glycerolipids in the yeast *Saccharomyces cerevisiae* Genetics 2012 15 317 349 10.1534/genetics.111.130286 22345606 PMC3276621

[B46] Hirst J King MS Pryde KR The production of reactive oxygen species by complex I Biochem Soc Trans 2008 15 976 80 10.1042/BST0360976 18793173

[B47] Bai RK Perng CL Hsu CH Wong LJC Quantitative PCR analysis of mitochondrial DNA content in patients with mitochondrial disease Ann N Y Acad Sci 2004 15 304 309 15126306 10.1007/978-3-662-41088-2_29

[B48] Kelly RDW Mahmud A McKenzie M Trounce IA St Joh JC Mitochondrial DNA copy number is regulated in a tissue specific manner by DNA methylation of the nuclear-encoded DNA polymerase gamma A Nucleic Acids Res 2012 15 10124 10138 10.1093/nar/gks770 22941637 PMC3488228

